# Total smoking bans in psychiatric inpatient services: a survey of perceived benefits, barriers and support among staff

**DOI:** 10.1186/1471-2458-10-372

**Published:** 2010-06-25

**Authors:** Paula Wye, Jenny Bowman, John Wiggers, Amanda Baker, Jenny Knight, Vaughan Carr, Margarett Terry, Richard Clancy

**Affiliations:** 1 School of Psychology, University of Newcastle, University Drive, Newcastle, 2308 Australia; 2 Hunter New England Population Health, Hunter New England Area Health Service, Longworth Avenue, Wallsend, 2287 Australia; 3 School of Medicine and Public Health, University of Newcastle, University Drive, Newcastle, 2308 Australia; 4 Centre for Brain and Mental Health Research, Faculty of Health, University of Newcastle, University Drive, Newcastle, 2308 Australia; 5 School of Psychiatry, University of New South Wales at St Vincent's Hospital, 390 Victoria Street, Darlinghurst, 2010 Australia; 6 Schizophrenia Research Institute, 405 Liverpool Street, Darlinghurst, 2010 Australia; 7 Mental Health Services, Hunter New England Area Health Service, The Mater Hospital, Edith Street, Waratah, 2298 Australia

## Abstract

**Background:**

The introduction of total smoking bans represents an important step in addressing the smoking and physical health of people with mental illness. Despite evidence indicating the importance of staff support in the successful implementation of smoking bans, limited research has examined levels of staff support prior to the implementation of a ban in psychiatric settings, or factors that are associated with such support. This study aimed to examine the views of psychiatric inpatient hospital staff regarding the perceived benefits of and barriers to implementation of a successful total smoking ban in mental health services. Secondly, to examine the level of support among clinical and non-clinical staff for a total smoking ban. Thirdly, to examine the association between the benefits and barriers perceived by clinicians and their support for a total smoking ban in their unit.

**Methods:**

Cross-sectional survey of both clinical and non-clinical staff in a large inpatient psychiatric hospital immediately prior to the implementation of a total smoking ban.

**Results:**

Of the 300 staff, 183 (61%) responded. Seventy-three (41%) of total respondents were clinical staff, and 110 (92%) were non-clinical staff. More than two-thirds of staff agreed that a smoking ban would improve their work environment and conditions, help staff to stop smoking and improve patients' physical health. The most prevalent clinician perceived barriers to a successful total smoking ban related to fear of patient aggression (89%) and patient non-compliance (72%). Two thirds (67%) of all staff indicated support for a total smoking ban in mental health facilities generally, and a majority (54%) of clinical staff expressed support for a ban within their unit. Clinical staff who believed a smoking ban would help patients to stop smoking were more likely to support a smoking ban in their unit.

**Conclusions:**

There is a clear need to more effectively communicate to staff the evidence that consistently applied smoking bans do not increase patient aggression. There is also a need to communicate the benefits of smoking bans in aiding the delivery of smoking cessation care, and the benefits of both smoking bans and such care in aiding patients to stop smoking.

## Background

Smoking remains responsible for the greatest disease burden in Australia [1] and elsewhere [2]. For those with psychiatric disorders, the prevalence of smoking [3,4] is much higher than among the general population [5]. In mental health inpatient settings in Australia [6,7] and elsewhere [8], high smoking rates have been reported. Consequently, those with a mental illness are more likely to develop and die from smoking-related diseases than are those without such an illness [9].

The introduction of total smoking bans represents an important step in addressing the harm caused by tobacco smoking for people with a mental illness [10]. The World Health Organisation [11] recommends that all health care premises and immediate surroundings should be smoke-free. Smoke-free workplaces in general not only protect nonï¿½smokers from the dangers of passive smoking [12], they also encourage smokers to quit or to reduce consumption [12-14]. Smoking bans are now the norm for indoor workers [15]. Total bans, where smoking is banned completely, are more effective than partial bans, where smoking is still allowed in designated areas [14].

Although general hospital settings have successfully moved to smoke-free environments [16], health services internationally are struggling with the challenges involved in implementing total smoking bans in mental health settings [17,18]. Evidence suggests that total smoking bans are more sustainable than partial smoking bans [10,19], more effective at reducing staff exposure to environmental tobacco smoke [19], and less likely to result in patient complaints or verbal aggression [10]. Additionally, the provision of nicotine dependence treatment is more likely to occur in psychiatric inpatient settings that adhere to total smoking bans [20,21]. Partial bans continue to condone smoking, sending a message that smoking is still an acceptable practice. In addition, partial bans do little to encourage patients to consider quitting, nor do they influence staff behaviour to provide nicotine dependence treatment [21,22]. This issue of changing staff and patient behaviour is a particularly important argument for total smoking bans [22-24].

The determinants of successful implementation of total smoking bans in mental health services have been suggested to include structural and systemic changes to health services, effective leadership, and staff acceptance [10,22,24]. While there is evidence that the implementation of a total smoking ban is accepted by the majority of mental health patients [10,19] and may increase patient optimism about success in quitting [10,25], a number of studies report negative staff views as barriers to the successful implementation of such bans [19,20,26]. Commonly reported staff-expressed barriers relate to fears of patient aggression [27], ethical concerns [19,20,28] and staff and patient compliance issues [19,20,26].

The ability to generalize from the findings of previous research regarding staff views towards smoking bans is limited by a number of methodological features and differences in methodologies between studies. Firstly, very few studies have examined staff support for smoking bans in mental health care settings that applied to both buildings and grounds; that is, a total smoking ban. Only four such studies were identified in the published literature over the last decade [14,20,29,30]. These four studies, undertaken in the Netherlands [20], the U.K. [29,30], and Switzerland [14], reported that 19%, 32%, 60% and 37% of psychiatric staff respectively were supportive of a total smoking ban. In contrast, studies of partial bans in mental health settings have reported much higher support [14,31].

Secondly, the four reported studies were conducted in different mental health settings. The Willemsen et al study involved aggregated data from psychiatric inpatient, outpatient, and sheltered home settings with a range of smoking restrictions in place [20], and the remaining studies were conducted in a variety of psychiatric unit settings [14,29,30]. The variety in reported levels of support for total smoking bans suggests differences in staff support for total smoking bans between different clinical settings.

Thirdly, despite the requirement for smoking bans to be complied with by all staff for the protection of all staff and patients, research regarding the acceptability of such bans has focused on the views of clinical staff only [14,20,22,24,26-31]. Given the different roles of clinical and non-clinical staff, and differences in the extent of contact with patients between such staff, the implementation of a smoking ban is likely to have different impacts on these staff groups, and hence likely to result in differences in perceived benefits, barriers and support. For the purposes of this study, 'clinical staff' is defined as those staff who in the course of their normal role, provided patient care.

Fourthly, the reported studies of total smoking bans were conducted at different times relative to the implementation of a total smoking ban. Two of the studies [14,29] indicated that measures of support for a total smoking ban were conducted following the implementation of such a ban. In contrast, the two studies[20,30] that reported the highest (60%) and lowest level of support for a total smoking ban (19%) respectively, were conducted prior to, and not in the context of an impending total smoking ban. Comparison of the reported levels of support between such studies is therefore of limited value, particularly as a person's response to change is influenced by the perceived personal consequence of the change, which in turn is influenced by the proximity or immediacy of the proposed change to the individual [32].

Further, two of the studies [14,29] reported significant levels of non-compliance following the implementation of a ban, as evidenced by respondent exposure to second hand smoke (35%) [14], and continued staff smoking (59%) [29]. The extent to which a smoking ban has been successfully implemented might be expected to impact on the extent and nature of perceived benefits, barriers and support for a smoking ban.

Despite the importance of identifying barriers and facilitators of staff support as a basis for designing the implementation of smoking bans, few studies have examined factors that are predictive of staff support for smoking bans. With respect to research specific to total smoking bans in psychiatric settings, only one study has done so, indicating that the support of Dutch clinical staff for a total smoking ban was positively associated with the belief that such a policy results in less annoyance from second hand tobacco smoke [20]. Similarly, a study to determine predictors of support for smoking bans among public officials found that support is significantly more prevalent among those who believe: that tobacco use is a serious problem in their community; breathing environmental tobacco smoke is a serious problem for non-smokers; government should get involved with people's decisions about smoking; in providing smoking-cessation programs for public employees; and have smoked less than 100 cigarettes during their lifetime [33]. No research has as yet been reported in Australia indicating staff support for either total or partial smoking bans in mental health settings.

This study was undertaken to determine clinical and non-clinical staff views regarding the imminent implementation of a total smoking ban in a large psychiatric hospital in Australia. The study had three aims: 1. to ascertain staff perceived benefits of, and barriers to a successful total smoking ban, 2. to investigate the level of staff support for total smoking bans within mental health services and their own units; and 3. to examine the association between clinician perceived benefits and barriers to a successful total smoking ban, and their support for such a ban in their unit.

## Methods

### Design & Setting

A cross-sectional survey of both clinical and non-clinical staff at a large psychiatric inpatient hospital in the state of New South Wales, Australia was undertaken. The facility had approximately 2000 patient discharges per annum, consisting of 80 beds in six units: a psychiatric emergency centre, an intensive care unit, two general acute units, a dual diagnoses (concurrent mental health and substance use) unit, and an aged care unit.

A 'smoke free workplace policy' that included a total smoking ban in both buildings and grounds was to be implemented in the facility two weeks immediately following the survey period. The introduction of the policy was in accordance with directions from the New South Wales Department of Health that all health facilities in the state were to become smoke-free. Preparations for the local implementation of the policy included: establishment of service-wide and mental health specific policy implementation committees; allocation of resources to the implementation of the policy; communication to staff and the community regarding the introduction of the policy; creation of a mental health implementation project officer position for twelve months; provision of quit and abstinence assistance for staff who smoked; placement of no smoking signage, removal of ashtrays, clinical staff consultations, and the provision of staff training.

### Participants

A total of 300 staff were employed at the site, of which 60% (approximately 180 staff) occupied clinical positions, that is, performed a role that involved patient care. The remainder occupied non-clinical positions (for example, administrative and support staff).

### Procedure

This research was approved by the Hunter New England Human Research Ethics Committee and the University of Newcastle Human Research Ethics Committee. Two questionnaires, one for clinical staff, and one for non-clinical staff were developed utilising concepts used in previous similar studies [22,30,34]. In addition, data from focus groups held with staff and ex-patients regarding a total smoking ban and nicotine dependence treatment guided the development of the questionnaires. Domains included identified issues to clinical practice change, and organisational policy objectives. Piloting of the survey with mental health clinical staff from other settings provided additional questions in the final version. The two questionnaires were very similar in content, with the clinical staff questionnaire including additional items relating to clinical care issues. All staff were invited by management email and staff newsletter to complete a pen and paper questionnaire during the two week survey period. Although completion of the questionnaire was voluntary, staff were encouraged to complete the questionnaire by management, and several prompts through emails and newsletters were provided. Questionnaires and return boxes were left in key locations (for example staff stations, tea rooms, reception areas) and research staff regularly checked each location during the survey period.

### Measures

#### Staff Demographic Characteristics

Demographic questions addressed respondent gender, age, highest level of education, job description, length of time in current position, current smoking status, and exposure to second hand tobacco smoke at work (yes, no) (Table 1). Clinical staff were also asked to indicate whether they had received training in smoking cessation care and whether they were interested in doing so.

**Table 1:** Respondent characteristics

	Clinical Staff (73)		Non-Clinical Staff (110)		Total (183)	
	%	n	%	n	%	N
Female	60	44	71	77	66	121
Age						
≤ 35	45	33	43	47	44	80
36 - 45	23	17	20	22	21	39
46+	32	23	37	40	35	63
Education						
< HSC	4	3	22	24	15	27
HSC	10	7	28	30	21	37
Undergraduate Degree	39	28	29	31	33	59
Postgraduate Degree	47	33	21	23	31	56
Length of Time in Job						
≤ 4 years	47	34	60	65	54	99
4+ years	53	39	40	44	46	83
Smoking Status						
Current smoker	19	14	23	25	21	39
Former smoker	33	24	24	22	26	48
Never smoker	48	35	55	60	52	95
Exposed to ETS at work	78	57	31	34	50	91
Trained in smoking care (yes)	19	13	na	na	na	na
Interested in such training (yes)	72	51	na	na	na	na

#### Perceived benefits of a total smoking ban

Both surveys contained a question which asked respondents to indicate their level of agreement with 14 statements regarding potential benefits of a total smoking ban ('strongly disagree, disagree, uncertain, agree, strongly agree') (Table 2).

**Table 2:** Perceived benefits of a total smoking ban

	Agree						Uncertain						Disagree					
	Clinical		Non Clinical		Total		Clinical		Non Clinical		Total		Clinical		Non Clinical		Total	
	%	n	%	n	**%**	**n**	%	n	%	n	**%**	**n**	%	n	%	n	**%**	**n**
Make the place look/smell better (183)	88	64	76	83	**81**	**147**	8	6	14	15	**11**	**21**	4	3	10	11	**8**	**14**
Help staff stop smoking (183)	67	49	65	71	**66**	**120**	18	13	27	29	**23**	**42**	15	11	8	9	**11**	**20**
Improve patient physical health (183)	69	50	62	68	**65**	**118**	17	12	27	29	**23**	**41**	14	10	11	12	**12**	**22**
Improve working conditions (183)	62	45	66	72	**64**	**117**	25	18	17	19	**20**	**37**	14	10	16	18	**15**	**28**
Increase patient quality of life (183)	41	30	39	43	**40**	**73**	33	24	42	46	**38**	**70**	26	19	18	20	**21**	**39**
Help patients stop smoking (183)	41	30	36	39	**38**	**69**	15	21	34	37	**29**	**52**	38	28	30	33	**33**	**61**
Increase the quality of care (183)	29	21	32	35	**31**	**56**	45	33	50	54	**48**	**87**	26	19	18	20	**21**	**39**
Improve patient mental health (183)	36	26	24	26	**29**	**52**	27	20	44	48	**37**	**68**	37	27	32	35	**34**	**62**
Make the unit safer (183)	26	19	27	29	**26**	**48**	31	23	39	43	**36**	**66**	42	31	34	37	**37**	**68**
Reduce medication use (72)	17	12					28	20					56	40				
Create less work (183)	18	13	8	9	**12**	**22**	33	24	39	43	**37**	**67**	49	36	52	57	**51**	**93**
Increase rapport between patients (72)	11	8					37	27					51	37				
Decrease client aggression (183)	10	7	7	8	**8**	**15**	33	24	30	33	**31**	**57**	57	42	62	68	**60**	**110**
Make patients happier (183)	3	2	7	8	**5**	**10**	37	27	35	38	**35**	**65**	60	44	58	63	**59**	**107**

#### Clinician perceived barriers to implementation of a total smoking ban

The survey for clinical staff included an item asking respondents to indicate their level of agreement with 19 statements regarding possible clinical barriers to a total smoking ban ('strongly disagree, disagree, uncertain, agree, strongly agree') (Table 3).

**Table 3:** Clinician perceived barriers to a successful total smoking ban

	Agree		Uncertain		Disagree	
	%	n	%	n	%	n
Fear of patient aggression	89	63	4	3	7	5
Patients will continue to smoke	72	52	14	10	14	10
Staff are too busy with patient mental health	61	43	15	11	24	17
Lack of staff cohesion/consistency	59	42	24	17	17	12
Staff resistance to change	58	41	22	16	20	14
Lack of staff time	57	41	21	15	22	16
Lack of staff confidence	53	38	21	15	26	19
Lack of staff knowledge	52	37	16	11	32	23
Staff will continue to smoke	51	37	24	17	25	18
Lack information about policy/procedures	49	35	21	15	30	21
Processes aren't developed	44	31	37	26	19	14
Support systems aren't in place	44	32	36	26	19	14
Lack of staff skills	43	30	14	10	43	30
Insufficient training provided	40	29	29	21	31	22
Lack of staff interest	36	26	26	19	38	27
Lack of resources	35	25	42	30	23	16
Lack of sustainability	32	23	32	23	36	26
Lack of management support	29	21	25	18	46	33
Lack of staff commitment	26	19	38	27	36	26

#### Support for a total smoking ban

Both questionnaires included a question asking whether the respondent supported a total smoking ban throughout all mental health services ('strongly unsupportive, unsupportive, no view either way, supportive, strongly supportive') (Table 4). The clinical staff survey also asked respondents to indicate their level of agreement with a total smoking ban in their unit ('strongly disagree, disagree, unsure, agree, strongly agree').

**Table 4:** Support for total smoking bans

	Clinical Staff		Non-Clinical Staff		All Staff	
	%	(n)	%	(n)	%	(n)
Do you support the statement that smoking should be totally banned throughout the Area's mental health services?^a^						
Strongly Unsupportive	10	7	6	6	7	13
Unsupportive	16	12	13	14	14	26
No view either way	10	7	13	14	12	21
Supportive	30	22	34	37	33	59
Strongly supportive	34	25	34	37	34	62
Do you agree with the statement that smoking should be totally banned on the unit^b^						
Strongly disagree	7	5				
Disagree	19	14				
Unsure	19	14				
Agree	22	16				
Strongly agree	32	23				

^a ^Clinical n = 73; Non-clinical n = 108; All n = 181

^b ^Clinical n = 72

### Analysis

All analyses were undertaken using SPSS Version 15 [35]. Descriptive statistics were used to report respondent demographics, perceived benefits of, and barriers to a total smoking ban, and support for a total smoking ban.

Response categories for staff perceived benefits and barriers were reduced to three: 'agree, uncertain, disagree'. Response categories for clinician and non-clinician support for a ban in mental health services generally were reduced to two: 'strongly unsupportive/unsupportive/no view either way'; and 'supportive/strongly supportive'. Response categories relating to clinician support for a ban in their unit were reduced to two: 'strongly disagree/disagree/unsure'; and 'agree/strongly agree'.

Possible differences between clinical and non-clinical staff in their perceptions of the benefits of a total smoking ban, and in their support for such a ban in mental health services generally were assessed by chi square analyses.

Chi square analysis was initially undertaken to determine the univariate associations between staff demographic characteristics and clinical staff perceptions of the benefits and barriers of a total smoking ban, and their support for such a ban. Multiple statistical testing was accounted for by setting the significance level to p < 0.01 [36,37]. Perceived benefits and barriers that had the strongest relationship with support for a total smoking ban were entered into a backward stepwise logistic regression model. The number of variables initially entered into the model was limited by the size of the sample. The final model contained all variables with p < 0.05.

## Results

### Participants

Of the 180 clinical and 120 non-clinical staff available to complete the survey, 183 (61%) did so: 73 clinical staff (41%), and 110 non-clinical staff (92%) (Table 1). Of the clinical respondents, 56% identified as nurses, 26% as allied health and 18% as medical/psychiatry Of the non-clinical respondents, 38% identified as administrative with patient contact, 30% as administrative with no patient contact, 14% as researchers, and 11% as hospitality staff (Table 1). Clinical staff were more likely to have a tertiary education (χ^2 ^= 26.033, p = .000) and to report being exposed to other people's smoke at work (χ^2 ^= 38.449, p = .000).

### Perceived benefits of a total smoking ban

The majority of respondents agreed that a total smoking ban would make their work environment look/smell better (81%) and improve working conditions (65%) (Table 2). Fewer agreed that a total smoking ban would improve their working environment in other respects, such as making the unit safer (27%), creating less work (12%) or decreasing client aggression (9%) (Table 2). While 66% agreed that a total smoking ban would help staff stop smoking, fewer (38%) agreed that it would do so for clients (Table 2). A majority agreed that a smoking ban would improve patients' physical health (65%), and 41% agreed that it would increase patient quality of life in general (Table 2).

A majority (69%) of respondents were either uncertain or did not agree that a total smoking ban would increase the quality of care overall, and 71% were either uncertain or did not agree that a total smoking ban would improve patient mental health (Table 2). In excess of 80% of respondents were either uncertain or disagreed that a total smoking ban would reduce medication use, increase rapport between patients or make patients happier (Table 2). There were no significant differences between the responses of clinical and non-clinical staff regarding the perceived benefits of a total smoking ban.

### Clinician perceived barriers to a total smoking ban

Details of clinicians' perceived barriers to a successful total smoking ban are provided in Table 3. Nearly all clinicians indicated a fear of patient aggression (89%), and most agreed that patients would continue to smoke (72%). More than half of the respondents indicated a lack of staff capacity to enforce the ban as a barrier to a successful total smoking ban, with such capacity being seen to be limited by staff resistance to change, and a lack of knowledge, confidence and skills among staff. In addition, a lack of organisational support in the form of information, training, processes, resources and systems was expressed by between 35% and 49% of clinicians (Table 3).

### Support for a total smoking ban

Levels of clinical and non-clinical staff support for total smoking bans within the mental health service generally, and the level of clinical staff support for such a ban in their specific unit are provided in Table 4. Approximately two thirds of both clinical (64%) and non-clinical respondents (68%) supported a total ban within mental health services generally, and 54% of clinicians supported the implementation of such a ban in their own unit (Table 4). The difference in the proportion of clinicians supporting a ban in mental health services generally, and the proportion supporting a ban in their own unit was statistically significant (p > 0.001).

Chi square analysis indicated no statistically significant differences in support for smoking bans in mental health services generally between clinical and non-clinical staff respondents.

### Association between clinician perceived benefits and barriers to a total smoking ban and their level of support for such a ban

Of the 9 demographic characteristics, and the 33 statements regarding clinician perceived benefits and barriers to a successful total smoking ban, 11 were found to be significant at p < .01 (Table 5).

**Table 5:** Association between clinician perceived benefits and barriers to a successful ban, and their support for such a ban in their unit

	clinicians that support a total ban	clinicians that do not support a ban	χ^2^	p
Smoker	4%	15%	7.503	.006
*Agreement with perceived positive impacts*		% agreement		
Improve patient physical health	45	24	8.829	.003
Improve working conditions	44	17	15.700	.000
Help staff stop smoking	44	22	9.063	.003
Help patients stop smoking	38	3	29.653	.000
Makes the unit safer	21	4	8.224	.004
Increase patient quality of life	32	8	12.365	.000
Improve patient mental health	29	6	13.730	.000
Increase the quality of care	25	4	11.885	.001
*Agreement with clinical issues of concern*				
Smoking bans aren't sustainable	10	22	7.667	.006
Lack of staff knowledge	37	16	8.713	.003

Logistic regression analysis results (Table 6) indicated that one variable was significantly associated with clinical staff support for a smoking ban in their own unit at p < .01. Respondents who believed a smoking ban would help patients to stop smoking were approximately 23 times more likely to support a smoking ban in their own unit than those who did not hold this view (p = .001).

**Table 6:** Results of the logistic regression analysis of the association between clinician perceived benefits and barriers to a total smoking ban and their support for such a ban in their unit

A total smoking ban in own unit	B	SE	df	p	Odds	Confidence Intervals	
						Lower	Upper
Will help patients stop smoking	3.140	.921	1	.001	23.107	3.801	140.473
Concerned that staff lack knowledge	1.738	.738	1	.018	5.688	1.340	24.154
Non-smoker	2.756	1.167	1	.018	15.739	1.598	154.987

## Discussion

This study has identified clear and consistent support for a total smoking ban in inpatient mental health services from a majority of both clinical and non-clinical staff, with little difference between these groups. Despite this support, and acknowledgement of the benefits for both staff and patients of such a ban, significant barriers to the success of a ban were expressed by staff, particularly with regard to fear of patient aggression and concerns regarding clinician capacity and organisational support for the ban. The only perceived benefit or barrier that was significantly associated with clinician support for a ban was the view that a ban would assist patients to quit smoking. These findings suggest that the introduction of total smoking bans can be supported by staff, but that maximizing such support is contingent on the implementation of strategies that address staff lack of knowledge of evidence regarding both the benefits of a smoking ban and the lack of a negative impact on patient behaviour, and of strategies that address staff capacity and provide organizational support for clinicians.

Although methodological differences between studies limit the ability to compare findings, the level of staff support observed in this study appears greater than that previously reported [14,20,29], and similar to that reported in the UK [30]. Temporal and jurisdictional differences may have contributed to the higher level of support observed in this study. In particular, the differences in outcomes may be attributed in part to this study being conducted in the context of a state-wide implementation of a smoke free policy in all health services, and of local strategies associated with its imminent implementation. To the extent that such a context contributed to the observed higher level of support, the findings confirm the previously reported importance of a systems approach to the successful implementation of a smoking ban specifically [21,38], and to organisational and clinical practice change generally [39].

The findings of a high prevalence of agreement by staff regarding the benefits of a total smoking ban on improving the workplace environment and working conditions are consistent with previous findings [20]. The results are also consistent with the findings that such improvements can be obtained following the implementation of total smoking bans in psychiatric facilities [40]. In contrast, the low levels of respondent agreement regarding possible benefits in terms of increased safety and decreased client aggression is not supported by evidence, as such benefits are able to be realized when a total smoking ban is implemented [10,19]. These latter findings suggest that a more effective communication of the broader range of benefits that can accrue from the implementation of a total smoking ban is needed. Education addressing the benefits of total smoking bans have been described as a key component in the sustainability of total smoking bans [24].

A similar pattern of findings was evident with regard to perceived benefits of a total smoking ban for patients. The finding that a majority of staff considered that the implementation of a ban would be beneficial for patient physical health is consistent with evidence suggesting such a benefit is possible [19,25]. However, the findings that a minority of staff considered that the implementation of such a ban would improve patient quality of life, mental health or help patients to stop smoking suggest that the potential benefits for patients are viewed quite narrowly. Research is required to determine whether these improvements for patients do occur.

Almost three quarters of staff agreed that patients would continue to smoke under a total smoking ban, a finding that is consistent with the findings of previous research that staff believe that patients will resist smoking bans [19,26]. Studies of patient views however indicate a degree of acceptance of smoking bans [19,25]. Studies of the effectiveness of total smoking bans on patient smoking behaviour have indicated that, although some patients continue to smoke after their implementation [10,29], smoking is less than prior to the implementation of a ban [14]. In a survey of forensic psychiatric inpatients' views, participants who smoked cited seeing staff and other patients smoking as barriers to quitting [41]. The advent of a total smoke-free environment offers an opportunity for changing tobacco use. This in itself is a step closer towards being a non-smoker, an opportunity that is unavailable in units where smoking is permitted [42,43]. In addition, without continuity of care and cessation support after discharge, smoking bans in inpatient settings risk having no long-term effect on patient quit attempts [44].

Almost all clinical staff (89%) expressed a fear of patient aggression. Evidence suggests that although this is a common concern among mental health staff [26-28], no significant increase in patient aggression occurs following the implementation of a total smoking ban [10,45-47]. More commonly, evidence suggests that following the implementation of such a ban an increase in patient [19] and staff support is evident [10,14,48-50], and levels of patient aggression are either unchanged or reduced [10,14]. This view may be related to a lack of skills or confidence in providing nicotine dependence treatment [24,26].

There was a low level of agreement that a total smoking ban would increase the quality of care overall. There was also little support for the view that a total smoking ban would improve patient mental health. These results may suggest a view that treating smoking is not valued as a clinical role, nor that it would benefit the health of patients. These findings suggests that considerable system change and staff support is required to provide an environment where a primary prevention approach such as nicotine dependence treatment can be sustained [22,24,51].

More than half of the respondents indicated a lack of knowledge, confidence and skills to provide care in the context of a total smoking ban, and between a third and half of respondents perceived a lack of organisational support in the form of the provision of information, training, organisational processes, systems and resources. This is a consistent finding in research regarding staff attitudes towards the implementation of smoking bans [10,20-22,24], and the provision of nicotine dependence treatment to mental health patients [42,44]. Such a consistency of findings suggests a clear need for health care services to implement smoking bans in a manner that includes strategies that address leadership, systems development, and staff training [21,22,24,42,44,51]. The finding that staff involved in this study expressed such views in the context of a system-wide approach to the implementation of a total smoking ban may indicate that the planned implementation strategies may not have been adequate, or that the impact of such strategies had yet to be experienced by staff. A follow up study of staff attitudes following the implementation of the smoking ban is required to determine the success of the implementation strategy in addressing these issues.

Only one perceived benefit or barrier was significantly associated with clinician support for a ban in their own unit - the belief that a ban would help patients to stop smoking. Such a finding suggests that clinicians are more likely to support a ban if they believe it will have a clinically beneficial outcome. Perhaps a potential strategy for encouraging staff support for a smoking ban in mental health inpatient settings is to emphasise the clinical and physical well-being benefits of a smoking ban, not just its environmental impacts, or the need for compliance with an organisational policy. The finding in this study of 69% of clinicians believing that a ban will improve a patient's physical health lends further support for such a proposal. Providing education and training to strengthen this believe may motivate staff to support the smoking ban [22,24,26], and skill staff to help patients cope with their concerns about tolerating a period of abstinence or a quit attempt.

The findings of the present study need to be considered in the context of a number of its methodological characteristics. First, although comparable to previous studies [20,29,30] the response rates, particularly for clinical staff, suggest that the results may not be representative of all staff. The extent to which the observed results reflect either an under or overestimate of the views of all staff is not known. Second, as the study was conducted in a single health service, the findings may not be generalisable to mental health services either elsewhere in the state or more broadly. However, a number of the findings appear consistent with the results of a previous study of the views of nurse unit managers across the state. For example, 69% of nurse unit managers state-wide [26] and 72% of clinicians in the current study report that patients aren't interested in quitting and will continue to smoke.

Although this was a study of staff views, further research is required to ascertain patient views towards total smoking bans. International research indicates that staff view a total smoking ban as more disruptive than patients [14]. For example, more staff than patients thought tobacco smoke was a source of conflict with staff or patients under a total smoking ban, and staff viewed co-habitation between smokers and non-smokers as more difficult under a total smoking ban [14]. Importantly, patients have reported to be more optimistic about being able to quit after an inpatient stay in a facility with a total smoking ban [25].

## Conclusions

Although it is acknowledged that implementing a total smoking ban in a setting that has an historical culture of acceptance of tobacco smoking will require significant attitudinal and system changes [21,24,26], strong leadership and staff training and support [24], there is a growing body of evidence to indicate that total smoking bans can be successfully implemented in psychiatric hospitals [10,18,24]. There is a clear need to more effectively communicate to staff the evidence that consistently applied smoking bans do not increase patient aggression, the benefits of smoking bans in aiding the delivery of smoking cessation care, and the benefits of both smoking bans and such care in aiding patients to stop smoking. The successful implementation of such bans in this setting is of particular importance as mental health treatment settings remain the only sector of health care that have failed to implement total smoking bans and systematically offer nicotine dependence treatment to patients. Such a failing perpetuates the health inequalities experienced by those with mental illness who smoke.

## Competing interests

The authors declare that they have no competing interests.

## Authors' contributions

All authors participated in the design of the study, development of the survey tool, and read and approved the final manuscript. PW completed data entry, data analysis, and drafting of the manuscript. PW, JB and JW were involved in critical revision of the manuscript.

## Pre-publication history

The pre-publication history for this paper can be accessed here:

http://www.biomedcentral.com/1471-2458/10/372/prepub
